# Experimental Validation of the New Modular Application of the Upper Bound Theorem in Indentation

**DOI:** 10.1371/journal.pone.0122790

**Published:** 2015-03-31

**Authors:** Carolina Bermudo, Francisco Martín, María Jesús Martín, Lorenzo Sevilla

**Affiliations:** Department of Civil, Material and Manufacturing Engineering, University of Malaga, Malaga, Spain; University of Zaragoza, SPAIN

## Abstract

Nowadays, thanks to the new manufacturing processes, indentation is becoming an essential part of the new arising processes such as the Incremental Forming Processes. This work presents the experimental validation of the analytical model developed for an indentation-based process. The analytical model is originated from the Upper Bound Theorem application by means of its new modular distribution. The modules considered are composed of two Triangular Rigid Zones each. The experimental validation is performed through a series of indentation tests with work-pieces of annealed aluminium EN AW-2030 and punches of steel AISI 304, under plane strain conditions. The results are compared with the ones obtained from the application of this new modular distribution of the Upper Bound Theorem, showing a good approximation and suitability of the model developed for an indentation-based process.

## Introduction

Indentation has been considered as a secondary manufacturing process [1] until now. Today, new manufacturing processes, such as the incremental forming processes (IFP), are being developed and indentation becomes an essential part of them. In the IFP, the work-piece final shape is obtained progressively. Since the dies used are smaller than the work-piece, the final shape is obtained gradually by repetitive applications of different impressions.

The IFP provides several advantages over the conventional manufacturing processes. As mentioned in [2] <<...*There are economical reasons to use incremental forming while there are also several technological advantages. In particular, the flexibility and the lower forces compared to die-defined forming are important as well as the properties and formability of the finished parts*...>>. Thanks to these advantages, the interest on incremental bulk processes is growing [3]. Consequently, the manufacturing industry demands new studies on indentation processes in order to perform more suitable applications according to their different perspectives.

In the indentation process, a die or punch produces an impression on the material or work-piece, causing permanent plastic deformation. If this action is consecutively repeated, different and complex shapes can be achieved. An implementation of the indentation process as a concatenation of indentations is exemplified by the Localized-Incremental Forming Processes (LIFP) [4]. Thus, LIFP is based on the sequence of specific compression processes. The work-pieces are larger than the tools or dies; therefore, global compression effects can be achieved in the work-piece by the concatenation of successive compression in predetermined positions. Multiple Indentation Processes (MIP) makes up another example of this. As explained in [5], <<...*In the multiple indentation process, a force is applied on the work-piece surface by a punch to produce an impression on the material. The compression process causes permanent plastic deformations that, repeated incrementally, let forming the work-piece by a localised-incremental forging action*...>> which can be compared with the shot-peening processes [6,7]. The LIFP and the MIP are considered as part of the so-called IFP [8]. This kind of processes requires less effort to achieve the needed deformation and the dies are, in general, geometrically simpler. Additionally, another advantage is that they can be entirely implemented in CNC machines with greater flexibility.

IFP are a recent alternative to traditional plastic deformation processes, such as forging or cold/hot rolling. These processes are referred to as traditional due to their wider development in the manufacturing industry. IFP processes arise in order to address the profound changes that are taking place in the industry, as a way to enable the current objectives of the modern industry. However, the industrial applications are still very restricted.

For this reason, as pointed out in [9], <<...*the demand for the simulation of incremental bulk forming processes is high. However, the computation times for the simulation of these processes are still unsatisfactorily long and thus, their application is deterred*...>>. Hence, a new modular distribution of the application of the Upper Bound Theorem (UBT), by means of modules of Triangular Rigid Zones (TRZ) is performed in this paper. This new method presents an alternative to the computational methods currently used in the industry, such as numerical methods or the Finite Element Method (FEM).

As can be seen, the study and analysis of IFP, where the indentation process takes place, is being a recurrent topic of research [10,11]. The implementation of these processes is becoming imperative to the industry in order to (be able to) offer a competitive production.

In this paper, IFP are approached as a set of successive indentations. In fact, the simpler case of study accounts as an indentation each time the punch compress the work-piece. This research reduces the analysis to an indentation case, showing the alternative resolution of the process by the optimized Upper Bound Theorem method (UBT).

In the study of the indentation process by the application of the UBT, the TRZ alternative is the kinematic-geometrical option [12] that allows reaching a more accurate solution and adds a strong capacity of analysis of the principal factors involved in the deformation process.

**Fig 1:**
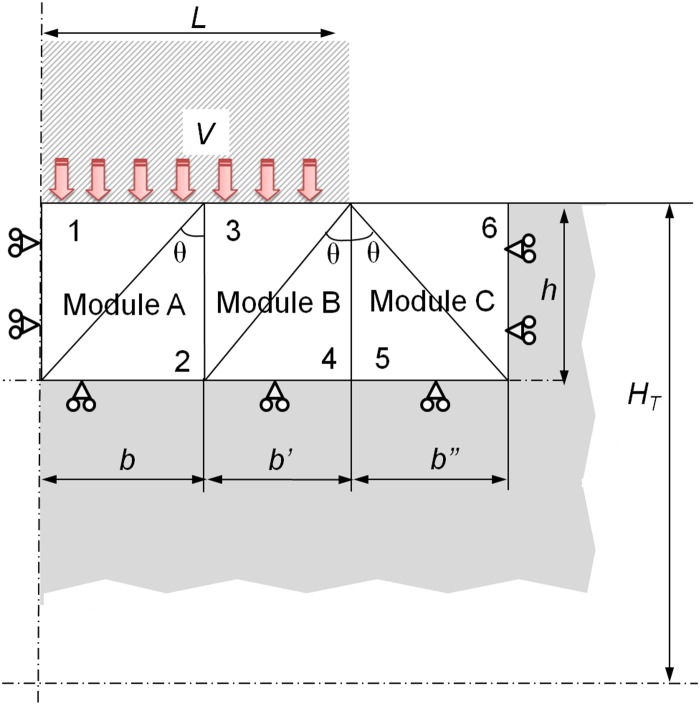
Optimal configuration.

The modular application of the UBT, implemented by F. Martín in 2009 [13], shows an improved approximation to the real effort values that are needed in the deformation processes studied [14,15]. After the appropriate model review for an indentation process, the optimal model is the one in Fig 1, being 2*L* the total width of the punch, *H*_*T*_ the total height of a quarter of the work-piece studied, *h* the modules, height, *V* the punch speed and *θ* the modules optimal angle.

Henceforth, the analytical study is focused only on a quarter of the work-piece involved in the deformation process. This is possible due to the double symmetry imposed on the analysis. The model in Fig 1 shows a modular configuration of three modules with two TRZ each. This configuration offers an easier analysis process thanks to the way it can be solved. The modular approach allows a resolution module by module, joining the final effort of each module in one final effort, conversely to the non-modular application of the UBT, which forces the entire solution of the model. The non-modular application presents a more complex resolution of the problem.

The analysis is proposed under Plane Strain conditions. Under this work restriction, the material offers its higher strength to deformation when *τ* = *k*, *τ* being the shear stress and *k* the yield strength in shear. Therefore, the value of the dissipative power, due to the internal energy, cannot exceed the outcome value of k·s·v*, *s* being the length that corresponds to the discontinuity line of the tangential velocity and v* the velocity in these discontinuities.

Also, under a plane strain hypothesis, the UBT, on general terms, expresses that <<... *The work realised by the superficial strengths of real traction (or compression) on a rigid-perfect plastic body is lower or the same that the realized by the superficial strengths of traction (or compression) corresponding to any other field of admissible kinematic velocities*...>> [16]

Then, Eq 1 shows its general expression

$$\int_{S_{v}}^{}T_{i}\cdot v_{i}\cdot {dS}_{v}\leq \int_{S_{D}}^{}k\cdot \left[v^{*}\right]\cdot {dS}_{D}+\int_{S_{F}}^{}T_{i}\cdot v_{i}^{*}\cdot {dS}_{F}$$ (1)

Being:

*T*_*i*_: External strengths applied on the work-piece;

*v*_*i*_: Real velocities field

*S*_*v*_: Surfaces where external loads are applied;

*S*_D_*: Discontinuity Surfaces

*v*_i_*: Kinematically admissible virtual velocities field

*S*_*F*_: External surfaces exposed to external surface stresses

Once the analytical model applicable under the UBT by its TRZ module configuration has been established and the final model optimization for the study of the indentation process implemented, a validation of the application through experimental testing, is necessary in order to determine the suitability of the method.

## Materials and Methods

### Preparation of the Test

**Fig 2:**
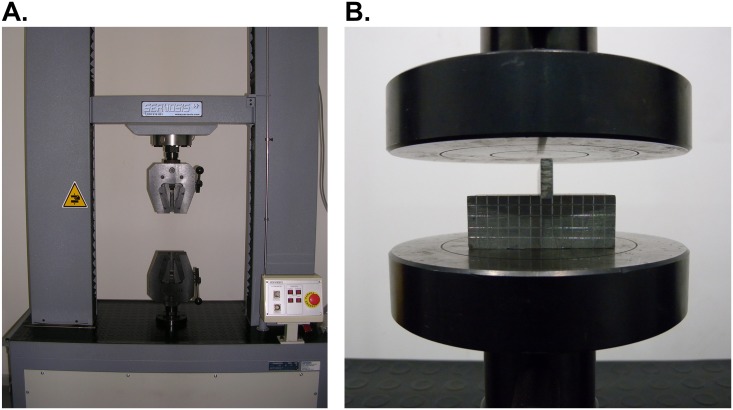
Preparation of the test. The tension-compression machine (A); and the compression plate tools used (B).

The trials were conducted with a tension-compression machine (Fig 2A and 2B) which was configured to apply continuous compression forces until the required indentation depth is achieved. This machine has a maximum load capacity of 100kN. Therefore, it is essential to work with materials endowed with wide range of deformation within the available strain values.

For the indentation tool, a 3 mm wide punch made of steel AISI 304is used. The work-piece is obtained from a 50x50x2000 mm square section bar of Aluminium EN AW-2030 (UNE-EN 573–1) [17]. The work-pieces final size is 50x50x30 mm, since the necessary depth to be able to work in Plane Strain conditions amounts to 30 mm. To achieve Plane Strain conditions, the work-piece depth should be at least 6 to 10 times the surface where the load is applied [18].

The material choice was made searching for a material commonly used in the industry. The aluminium alloy considered is often used in the aircraft structures manufacture, usually after having been subjected to different thermal-mechanical treatments. The principal alloying element in these aluminium alloys is copper. Previous tests where made with lead and tin due to their higher ductility and lower effort requirements to accomplish the expected plastic deformation. The suitability of the trial was analysed through the former tests; consequently, a special tool for the punch was designed. The analysis revealed that, at high velocities, the punch tended to tilt.

**Fig 3:**
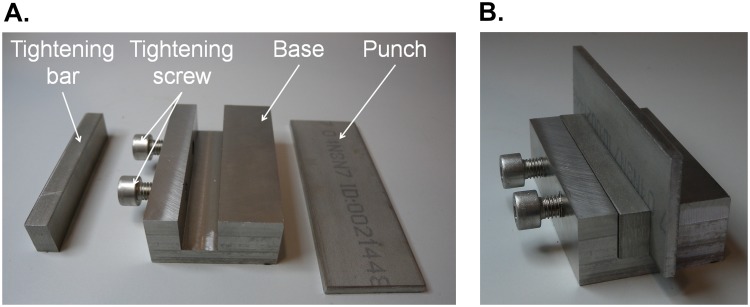
Punch fastening tool. Tool parts for the punch fastening (A); and the tool assembled (B).

Thus, a clamp system was needed. A stabilizing system for the indenter (Fig 3A) was manufactured to prevent the slippage of the punch along the compression plates and achieve a complete and proper deformation of the work-piece. The designed tool is composed of two clamping screws that push a horizontal steel bar, called clamping bar, which, in turn, performs a uniform pressure over the indenter. This support (Fig 3B) provides a wider base to the indenter, avoiding leaning.

**Fig 4:**
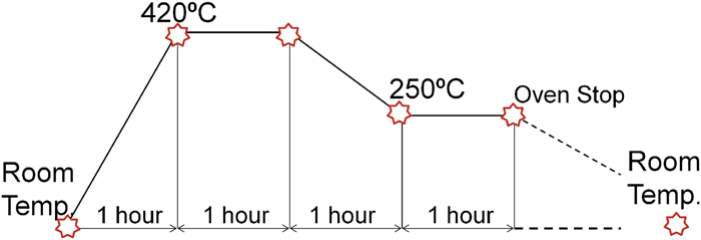
Annealing cycle.

To achieve an adequate depth during the indentation test by providing strain values lower or equal to 100 kN, the aluminium work-pieces need to be processed. The material is subjected to a controlled annealing treatment (Fig 4), thereby attaining aluminium work-pieces with different properties from the initial work-pieces subtracted from the aluminium bar. This new aluminium state allows bigger deformations with lower efforts so that it can be plastically deformed with ease using the available tension-compression machine.

**Fig 5:**
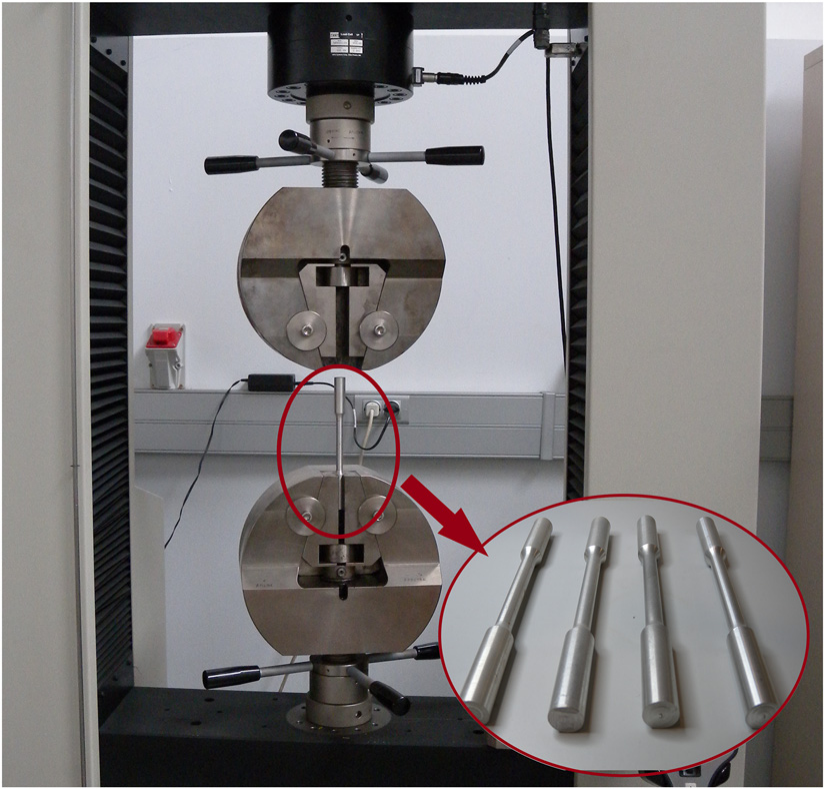
Tensile test. Tensile samples used for the tensile test.

In addition to this, the correct implementation of the new modular distribution of the UBT requires knowing both, the strength coefficient *K* and the strain-hardening (or work-hardening) exponent *n* of the new aluminium obtained after the annealing process. Since the characteristics of the aluminium obtained after the annealed treatment are different from the one considered before and consequently the *n* and *K* coefficients are also different and unknown, a series of tensile tests are implemented in order to acknowledge them (Fig 5). To this effect, a set of tensile samples (Fig 5) are manufactured according to the UNE-EN ISO 6892–1 [19] (Table 1) which undergo the same cycle annealing as the work-pieces.

**Table 1:** Standard cross-sectional specimens (UNE-EN ISO 6892–1).

Proportionality coefficient	Diameter (mm)	Initial length between marcs	Minimum length of the calibrated part (mm)
c	*d*	*$L_{o}=c\sqrt{S_{o}}$*	*L*_*c*_
5.65	20	100	110
5.65	14	70	77
5.65	10	50	55
5.65	5	25	28

**Fig 6:**
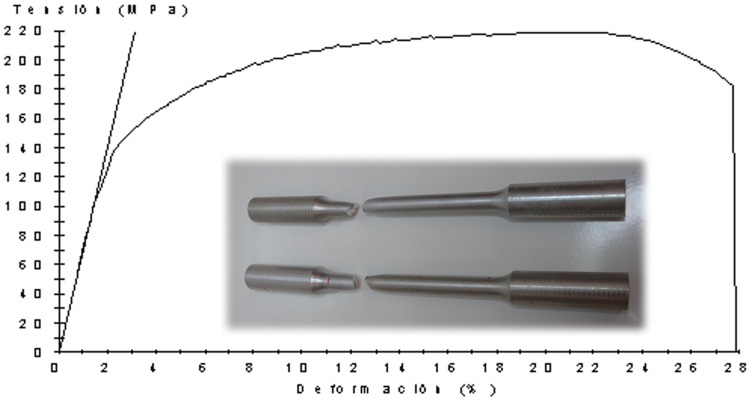
Tested samples. Stress-strain curve and tensile samples after tensile test.

After the tensile test is conducted (Fig 6), the stress-strain curve can be plotted. By applying the standard E 646–00 [20] used for the determination of *n* and *K*, each value is obtained for aluminum EN AW-2030 after annealing. These values are *n* = 0.26 and *K* = 404.66 MPa. Eventually, implement the characteristics of the material in the UBT analysis and obtaining the efforts needed to achieve the plastic deformation specified is possible.

### Indentation Test

**Fig 7:**
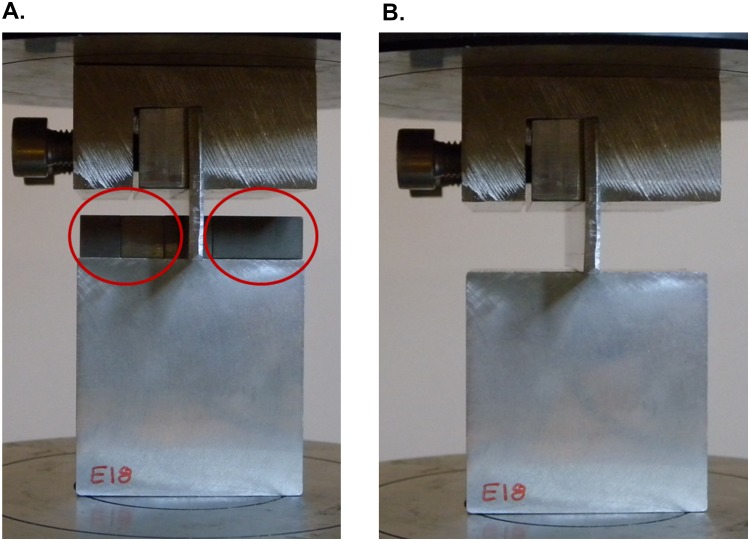
Indentation test. Centered punch (A); and Pre-load punch before the indentation test (B).

In order to complete the indentation test, ensuring the centering of the punch during the whole process is essential. For this reason, block patterns are used to guarantee the correct position (Fig 7A). After securing the indenter with a pre-load of 50 Newton, the block patterns can be removed (Fig 7B).

Once the whole system is established, an indentation of 6 mm is conducted. The indentation depth cannot be increased to avoid excessive damage of the work-piece during deformation. Knowing that overcome the tensile strength limit is not intend, an excessive damage is considered when cracks start to appear in the work-piece.

In order to establish the speed influence on the work hardening effect of the selected material, various tests with different strain rates were performed. To obtain a right mean of results, three trials are carried out for each studied speed, 4 mm/min and 60 mm/min, as well as two trials for extreme speeds, 400 mm/min and 0.6 mm/min in order to study the material behavior in a wider range of speeds.

Each tested work-piece was given a code for identification, as follows:

$$EX_{1}-X_{2}-X_{3}-A_{1}-X_{4}$$

Being:

*A*_1_: Material of the specimen

X1: Number of specimen

X2: Speed (mm/min)

X3: Depth of the indentation

X4: Dimensions of the specimen (mm)

The main performed tests are presented in Table 2.

**Table 2:** Test performed.

A1	X1	X2 (mm/min)	X3	X4 (mm)
Annealed Al. EN AW-2030	E19	4	6	50x50x30
Annealed Al. EN AW-2030	E20	4	6	50x50x30
Annealed Al. EN AW-2030	E21	4	6	50x50x30
Annealed Al. EN AW-2030	E22	60	6	50x50x30
Annealed Al. EN AW-2030	E23	60	6	50x50x30
Annealed Al. EN AW-2030	E24	60	6	50x50x30

### Upper Bound Approach

Regarding the UBT approach, only the Infinite case has being considered (Fig 1), since the configuration of the Finite case analyzed in previous studies [21] is customarily identified with processes that differ from indentation and are more focused on sheet metal treatment, consequently remaining out of the scope of this study.

Thanks to the modular consideration, the analysis can be performed module by module. A weighted average is carried out to obtain the final values of *p*/*2k*. (Eq 2)

$${\left(\frac{p}{2k}\right)}_{T}\;=\;\frac{{\left(\frac{p}{2k}\right)}_{A}\cdot b{+\left(\frac{p}{2k}\right)}_{B}\cdot b^{'}{+\left(\frac{p}{2k}\right)}_{C}\cdot b^{''}}{b+b^{'}+b^{''}}$$ (2)

Where *p* is the effort required to deform the work-piece and *b*, *b*', *b*'' are the widths of the bases corresponding to the modules A, B and C, respectively.

**Fig 8:**
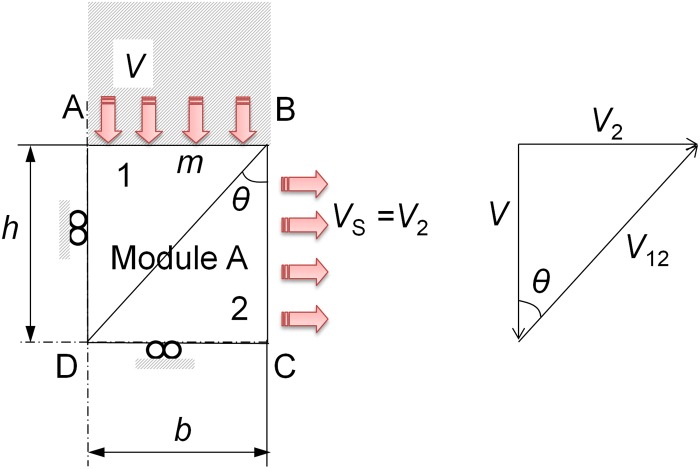
Module A analysis.

The analysis starts with Module A considering it only receives the vertical thrust of the punch. Module B receives the thrust of the punch and the previous module (A), while the external Module C only receives the thrust of Module B. The approach of the analysis and hodograph for the first module is shown in Fig 8.

Solving the UBT for Module A as shown in Eq 3, the *p*/2*k* expression for Eq 4 can be obtained as follows:

p·V·b·w = k·w·V1·AB·m+V12·BD+V2·DC = k·Vcosθ·hcosθ+V·tgθ·b = k·1hb2+h2·hhb2+h2+b2h (3)

$${\left(\frac{p}{2k}\right)}_{A}=\frac{1}{2b}\cdot \left[\frac{h^{2}+2b^{2}}{h}\right]$$ (4)

### Hardening

Finally, to incorporate the hardening effect that take place as a result of the deformation process discussed in this article, the Ludwik equation (Eq 5) is used, and incorporated in the model as shown in Table 3.

**Table 3:** Hardening Model selection according to *n*.

Material	*n*	Hardening Model
Aluminium	0 ≤ *n* ≤ 0.10	3
Aluminium	*n* > 0.10	2

$$\sigma \;=\;Y+K\cdot \varepsilon^{n}$$ (5)

Where *σ* is the yield stress to overcome the needed deformation, *Y* is the yield strength of the material at the initial point and *ε* the plastic strain. Performing the appropriate substitutions Eq 6 raises.

$$k\;=\;k_{o}+0.6\cdot K\cdot \varepsilon^{n}$$ (6)

Therefore, for each stage of deformation considered, *k* will increase according to the previous one.

The difference between the Hardening Models (HM) used lies in the number of modules in which the hardening effect is considered (Table 3). So, for HM 1, the hardening is applied to the 3 modules alike, for HM 2 it is applied to those below the punch, A and B, while for HM 3 it is only applied to the first module, A.

According to this classification, the appropriate model for the material being tested was HM 2.

## Results and Discussion

Thanks to the annealing process carried out, an indention process with the aluminium EN AW-2030 work-pieces is possible. The depth achieved is far from the range of application of the conventional hardness tests but closer to current manufacturing processes such as the IFP.

**Fig 9:**
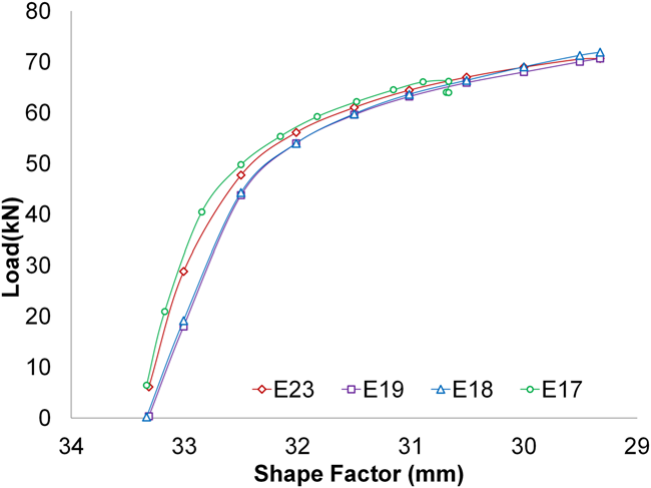
Efforts. Efforts obtained using different speeds.

A series of tests using different speeds have been made to check how the speed parameter affects the hardening of the specimen. The ratios tested were 400 mm/min, 0.6 mm/min, 4 mm/min and 60 mm/min. Fig 9 shows the results of the tests E17-400-6-Al2030O-50x50x30, E18-0.6-6-Al2030O-50x50x30, E19-4-1.5-Al2030O-50x50x30-I and E23-60-6-Al2030O-50x50x30. It can be appreciated that the speed does not produce significant changes in the resulting stresses. The shape factor present in the plotted graphics corresponds to *H*_*T*_/*L*.

**Fig 10:**
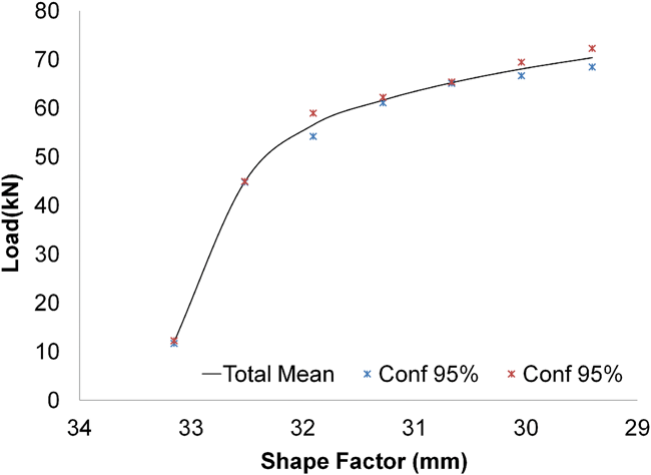
Confidence interval.

To support the null hypothesis, a statistical study was performed following the UNE 66040:2003 standard (ISO 2602:1980 equivalence), “Statistical interpretation of test results. Mean Estimation. Confidence interval” [22]. Fig 10 shows the confidence interval that results from comparing the tests performed at different speeds, being this confidence interval of 95%.

**Fig 11:**
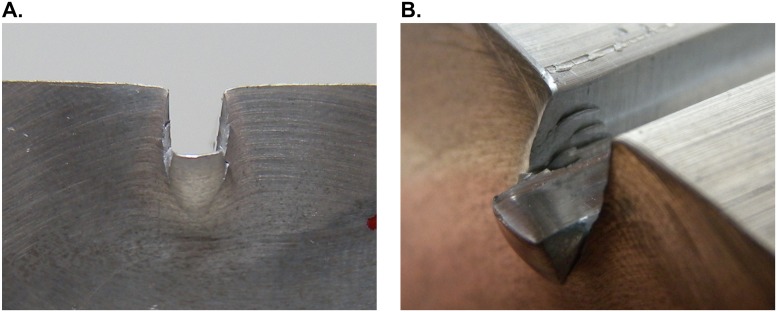
Indented sample. Indentation performed (A); and detail of the samples cracks (B).

Fig 11 displays the test performed on the work-piece E22 at a speed of 60 mm/min (Fig 11A) and a closer look to the indentation caused (Fig 11B). As can be seen, certain fragility in the material is appreciated. Cracks appear in the surface of the work-piece near the margins of the punch. This phenomenon may distort the results for a later comparison, justifying that deeper indentations are undesirable.

**Fig 12:**
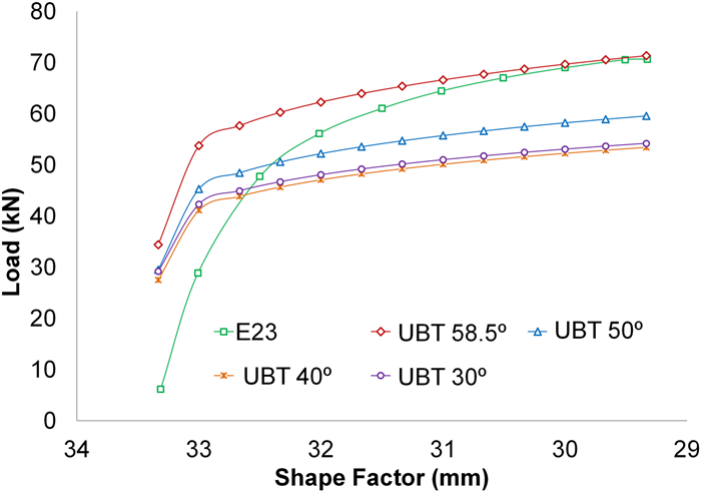
Results. Results evolution depending on the angle for E23.

In Using the modular distribution studied during the application of the UBT, the optimal results are obtained for an angle of *θ* = 58.5°. Therefore, the highest difference between the results obtained applying the analytical method and those obtained from the tests is reached at/for the first values of the shape factor, as can be observed in Fig 12. This first part of the graph relates to the first instants of the test. Once exceeded 1 mm in the indentation, the results begin to converge, showing a proper evolution thereof and a good performance of the model set.

**Fig 13:**
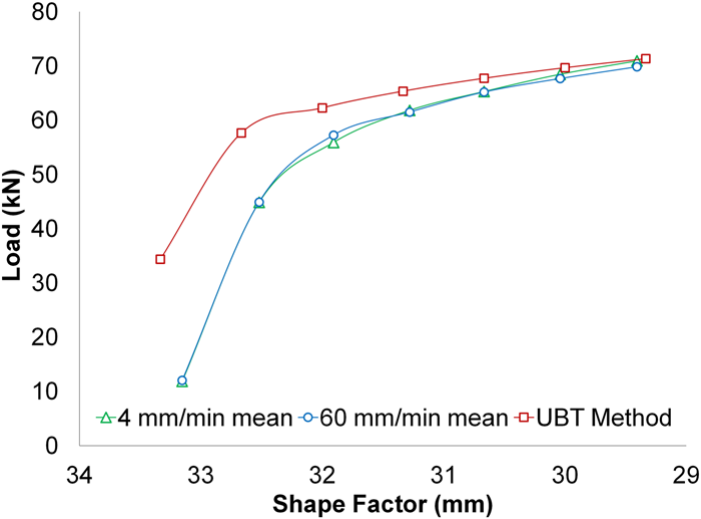
Comparative between test and UBT model.

Ultimately, to develop a comparative study between the tests conducted and the results accomplished with the model of the UBT new modular distribution, a mean average for the test groups of 60 mm/min, and 4 mm/min is performed. This comparison is shown in Fig 13. The UBT modular model follows the evolution of the stresses obtained in the indentation tests carried out.

As the indentation gains depth, the difference between the developed analytical model and the results obtained with the experimental tests decreases. Taking into account that shallow indentations (1 or 2 mm) are not interesting from this study’s point of view, the first values of the graph can be disregarded. Hence, with a 2 mm indentation, the difference between the modular UBT model and the test at 4 mm/min and 60 mm/min are of 11.4% and 8.8% respectively. Thus, the deeper the indentation gets, the lower this difference is. Finally, at a 6 mm indentation the differences are become 0.6% and 2.1%. These results are consistent with other studies that apply the UBT to different manufacturing processes and compare results with experimentation [23–27].

## Conclusions

In this paper, the experimental validation analysis of the application of the Upper Bound Theorem in indentation by its new modular configuration is presented. This new configuration is based on modules of two Triangular Rigid Zones each. Indentation is considered to be included within the new processes that are been developed in the field of forging, such as the Incremental Forming Processes.

Only the Infinite consideration of the model has been studied, since this is more related to indentation processes. Of the two configurations, the Infinite one is admitted as the general application. The Infinite configuration covers the range of work-pieces normally used in the manufacturing industry for forming processes such as indentation. The range of finite work-pieces resembles the deformation done by sheet metal forming processes, which are out of the scope of this study. In order to make a comparison of the results obtained with the new modular model of the Upper Bound Theorem, a substantial number of indentation tests have been raised, assuming plane strain. Moreover, the influence that the strain rate has on the strain hardening parameter has been studied, having noticed that, for the different speeds studied, the results show similar values. Therefore, in the cases studied, the influence of the speed on the strain hardening effect can be rejected, due to the Infinite consideration of the work-piece.

Making the comparison between the mean values of the tests performed, for the different speeds considered, and the results obtained applying the new modular consideration of the Upper Bound Theorem, the similarity in the evolution of both analysis can be appreciated. There is a wide difference in the early stages of the deformation process (between 0 and 1 mm of penetration); however, the model tends to stabilize the results showing a progressively approach to the real effort values. The optimal approach is obtained with an angle of 58.5° for the selected modular model.

Thereby, a method that simplifies the existing methods for the study of the efforts needed in deformation is presented. On the one hand, from the mathematical perspective, the application of the Upper Bound Theorem under its modular configuration and after the model is properly developed, involves a less complicated implementation compared to other methods, such as the Finite Element Method. On the other hand, from the analytical perspective, the modular consideration simplifies the mathematical resolution of the problem, presenting a simpler final equation.

Similarly, its lower computational cost makes the new developed method appropriate to achieve the study of the efforts needed to carry out the relevant deformation. In addition, thanks to the versatility of the modular configuration exposed, the adaptation of the model to different dies geometries is possible.

The possibility to incorporate different energy factors involved in the forming process is demonstrated. This enables the discrimination of each energy factor, being able to determine their influence on the deformation process, as it was carried out with the hardening effect. Likewise, the inclusion of the different parameters that can be present in a deformation process, allows working with a model closer to reality.

Finally, with the developed method it is possible to establish the efforts curves of the deformation process. Thus, the upper limit necessary to ensure the deformation can be obtained.

## References

[1] Chakrabarty J. Theory of Plasticity. U.K.: Elsevier Science; 2006.

[2] Groche P, Fritsche D, Tekkaya EA, Allwood JM, Hirt G, Neugebauer R. Incremental Bulk Metal Forming. CIRP Ann-Manuf Technol. 2007;56: 635–656.

[3] Allwood JM, Utsunomiya H. A survey of flexible forming processes in Japan. Int J Mach Tools Manu. 2006;46: 1939–1960.

[4] Camacho AM, Marín MM, Rubio EM, Sebastian MA. Application of different simulation strategies for the analysis of Multi-Stroke Localised-Incremental Forming operations. Advances in Non Conventional Materials Processing Technologies. Mater Sci Forum. 2012;713: 19–242.

[5] Bernal C, Camacho AM, Marín M, de Agustina B. Methodology for the evaluation of 3D surface topography in Multiple Indentation Processes. Int J Adv Manuf Tech. 2013;69: 2091–2098.

[6] Jianming W, Feihong L, Feng Y, Gang Z. Shot peening simulation based on SPH method. The Int J Adv Manuf Tech. 2011;56: 571–578.

[7] Bernal C, Camacho AM, Arenas JM, Rubio EM. Analytical procedure for geometrical evaluation of flat surfaces formed by Multiple Indentation Processes. App Mech Mater. 2012;217–219: 2351–2356.

[8] Nowak J, Madej L, Grosman F, Pietrzyk M. Material flow analysis in the Incremetal Forging technology. Inter J Mater Form. 2010;3: 931–934.

[9] Hirt G, Kopp R, Hofmann O, Franzke M, Barton G. Implementing a high accuracy Multi-Mesh Method for incremental Bulk Metal Forming. CIRP Ann-Manuf Technol. 2007;56: 313–316.

[10] Alfozan A, Gunasekera JS. Design of profile Ring Rollin by Backward Simulation using Upper Bound Element Technique (UBET). J Manuf Process. 2002;4: 97–108.

[11] Bagudanch I, Garcia-Romeu ML, Ferrer I, Lupiañez J. The effect of process parameters on the energy consumption in Single Point Incremental Forming. Procedia Eng. 2013;63: 346–353.

[12] Topcu N. Numerical, analytical and experimental analysis of Indentation. Ph.D. Thesis, Turkish: Midlle East Technical University. 2005. Available: etd.lib.metu.edu.tr/upload/12605942/index.pdf

[13] Martín F, Camacho AM, Domingo R, Sevilla L. Modular procedure to improve the application of the Upper-Bound Theorem in Forging. Mater Manuf Process. 2013;28: 282–286.

[14] Rubio EM, Domingo R, Arenas JM, González C. Energetic analysis of the drawing process by Upper-Bound Techniques. J Mater Process Tech. 2004;155–156: 1220–1226.

[15] Martín F, Sevilla L, Sebastian Perez MA. Implementation of technological and geometrical parameters in forging processes by means of the Upper Bound Element Technique. AIP Conf Proc. 2009;1181(1): 455–463.

[16] Johnson W, Mellor PPB. Engineering Plasticity. Chichester:Ellis Horwood Limited; 1983.

[17] UNE-EN 573–3. Aluminium and aluminium alloys—Chemical composition and form of wrought products Part 1: Numerical designation system. Madrid AENOR; 2005.

[18] Rowe GW. An Introduction to the Principles of Metalworking. California: Edward Arnold Limited; 1971.

[19] UNE-EN ISO 6892–1. Metallic materials—Tensile testing Part 1: Method of test at room temperature (ISO 6892–1:2009). Madrid AENOR; 2010

[20] E 676-00 Standard Test Method for Tensile Strain-Hardening Exponents (n-Values) of Metallic Sheet Materials. West Conshohocken: ASTM International; 2007

[21] Bermudo C, Martín F, Sevilla L. Analysis and selection of the modular block distribution in indentation process by the Upper Bound Theorem. Procedia Eng. 2013;63:388–396.

[22] UNE 66040. Statistical interpretation of test results. Estimation of the mean Confidence interval. Madrid AENOR; 2003.

[23] Altinbalik T, Ayer O. Effect of die inlet geometry on extrusion of clover sections through curved dies: Upper Bound analysis and experimental verification. T Nonfer Metal Soc. 2013;23: 1098–1107.

[24] Hwang BC, Hong SJ, Bae WB. An UBET analysis of the non-axisymmetric extrusion/forging process. J Mater Process Tech. 2001;111: 135–141.

[25] Hwang BC, Lee HI, Bae WB. A UBET analysis of the non-axisymmetric combined extrusion process. J Mater Process Tech. 2003;139: 547–552.

[26] Parvizi A, Abrinia K. A two dimensional Upper Bound Analysis of the ring rolling process with experimental and FEM verifications. Int J Mech Sci. 2014;79: 176–181.

[27] Khoddam S, Farhoumand A, Hodgson PD. Upper-Bound analysis of axi-symmetric forward spiral extrusion. Mech Mater. 2011;43: 684–692.

